# High Concentration Hydrogen Mitigates Sepsis-Induced Acute Lung Injury in Mice by Alleviating Mitochondrial Fission and Dysfunction

**DOI:** 10.3390/jpm13020244

**Published:** 2023-01-29

**Authors:** Nan Zhao, Ruiqiang Sun, Yan Cui, Yu Song, Wanjie Ma, Yingning Li, Jing Liang, Guolin Wang, Yonghao Yu, Jiange Han, Keliang Xie

**Affiliations:** 1 Department of Anesthesiology, Tianjin Institute of Anesthesiology, Tianjin Medical University General Hospital, Tianjin 300052, China; 2 Department of Anesthesiology, Tianjin Chest Hospital, Tianjin 300308, China; 3 Department of Anesthesiology, Tianjin Eye Hospital, Tianjin 300020, China; 4 Department of Pathogen Biology, School of Basic Medical Sciences, Tianjin Medical University, Tianjin 300070, China; 5 Department of Critical Care Medicine, Tianjin Medical University General Hospital, Tianjin 300052, China

**Keywords:** sepsis, acute lung injury, high concentration hydrogen, mitochondrial dysfunction, antioxidant

## Abstract

*Background*: Multiple organ failure (MOF) is the main cause of early death in septic shock. Lungs are among the organs that are affected in MOF, resulting in acute lung injury. A large number of inflammatory factors and stress injury in sepsis can lead to alterations in mitochondrial dynamics. Numerous studies have confirmed that hydrogen can alleviate sepsis in the animal model. The purpose of this experiment was to explore the therapeutic effect of high concentration (67%) hydrogen on acute lung injury in septic mice and its mechanism. *Methods*: The moderate and severe septic models were prepared by cecal ligation and puncture. Hydrogen with different concentrations was inhaled for one hour at 1 h and 6 h after the corresponding surgery. The arterial blood gas of mice during hydrogen inhalation was monitored in real time, and the 7-day survival rate of mice with sepsis was recorded. The pathological changes of lung tissues and functions of livers and kidneys were measured. The changes of oxidation products, antioxidant enzymes and pro-inflammatory cytokines in lungs and serums were detected. Mitochondrial function was measured. *Results*: The inhalation of 2% or 67% hydrogen improves the 7-day survival rate and reduces acute lung injury as well as liver and kidney injury in sepsis. The therapeutic effect of 67% hydrogen inhalation on sepsis was related to increasing antioxidant enzyme activity, reducing oxidation products and pro-inflammatory cytokines in lungs and serums. Compared with the Sham group, mitochondrial dysfunction was alleviated in hydrogen groups. *Conclusions*: Hydrogen inhalation by high or low concentration can both significantly improve sepsis; however, a high concentration demonstrates a better protective effect. High concentration hydrogen inhalation can significantly improve the mitochondrial dynamic balance and reduce the lung injury in septic mice.

## 1. Introduction

Sepsis is the second leading cause of mortality among patients in non-coronary intensive care units, which may lead to life-threatening organ dysfunction [1]. Sepsis is a host immune response caused by infectious factors, including fungi, bacteria and viruses. It is a syndrome dominated by systemic inflammatory response. Severe sepsis is closely related to the increase in short-term mortality. At present, studies have confirmed that the general condition of patients with severe sepsis deteriorates rapidly and makes them enter a state of immunosuppression, resulting in various immune-related complications [2]. The mechanism of the systemic inflammatory response in sepsis is very complex. Inflammatory response in a certain intensity and range is beneficial to the body, but it may cause serious damage to the body if it exceeds a certain limit. When sepsis occurs, the patient could develop an acute lung injury (ALI) or acute respiratory distress syndrome (ARDS).

So far, studies have demonstrated that mitochondrial dysfunction plays an important role in the pathogenesis of sepsis [3,4,5,6]. Mitochondria, the organelles that produce the energy required by human body, play a key role in the whole life activities of human body. ATP produced by mitochondrial oxidative phosphorylation provides 95% of the energy needed for cell activity [7]. The integrity of the mitochondrial structure and function is very important for the normal operation of the whole body. In addition to the ATP production, mitochondria have many other functions, including regulating the intracellular calcium content, producing reactive oxygen species (ROS), regulating cell signal transduction, redox processes, etc. The structure of the mitochondria is highly dynamic. The shape of it is unique to cell types and can be modified according to the changes of energy production, calcium homeostasis, lipid biogenesis, fatty acid synthesis and other activities. Mitochondrial morphological change is achieved by regulating its dynamic characteristics, including fusion, fission, movement and positional tethering. Mitochondrial dynamics also play an important role in a variety of cellular signaling pathways. Therefore, many mechanisms have evolved in regulating its structure. These regulatory mechanisms are important for regulating specific processes of mitochondria and the response of cells, tissues and organisms to developmental or environmental signals [8]. A large number of inflammatory factors and stress injury in sepsis can lead to changes in mitochondrial dynamics and affect the processes of its division, fusion and autophagy.

The effect of oxidative stress on sepsis has long been expounded and demonstrated by many scholars. Analysis demonstrated that 2~4% hydrogen gas (H_2_) or hydrogen-rich saline (normal saline containing a therapeutic dose of hydrogen) could eliminate toxic oxygen free radicals in animals to a certain extent, and thus exert antioxidant function [9]. In recent years, there are more and more studies on the use of hydrogen in the medical field, and it has been widely recognized as a new medical gas molecule [2,10]. At present, a large number of studies have confirmed that hydrogen inhalation or hydrogen-rich water can improve the mortality of the sepsis animal model, significantly reduce the damage of important organs and improve organ function. In recent years, with the application of the new high concentration hydrogen treatment equipment, there are more and more studies on the treatment of various diseases with high concentration hydrogen (67%), and good clinical effects have been achieved [11]. Theoretically, high concentration hydrogen inhalation can increase the hydrogen concentration in tissues, cells and even organelles and enhance the antioxidant effect. Whether the effect of high concentration hydrogen in the treatment of sepsis is better than that of low concentration hydrogen, and the specific mechanism of the therapeutic effect of high concentration hydrogen, need to be further confirmed and studied.

Therefore, this experiment aimed to explore whether high concentration hydrogen inhalation can effectively improve the functional injury of important organs in moderate and severe sepsis mice, and whether high concentration hydrogen inhalation can provide protection against the sepsis-induced lung injury by improving the mechanism of mitochondrial dynamics.

## 2. Materials and Methods

### 2.1. Animals

Adult male C57BL/6J mice, weighing 20 g–25 g, were provided by the experimental animal center of the Chinese Academy of Military Medical Sciences. Mice were raised in an environment without special pathogens: the temperature was 20 °C–22 °C, the humidity was about 40%, 12 h a day, alternating day/night, free to eat and drink. All experimental procedures were approved by the experimental animal management committee of Tianjin Medical University.

### 2.2. Cecal Ligation and Puncture (CLP)

Mice were anesthetized by intraperitoneal injection of 1% pentobarbital sodium (50 mg/kg). After the mice had no response to external stimuli, their limbs were fixed in the test position. Abdominal hair removal and skin preparation: iodophor disinfection, sterile gauze coverage, a 1 cm incision in the center of the abdomen, and separated into the abdominal cavity according to the anatomical level. The cecum in the abdominal cavity was explored with smooth forceps. Mouse model with moderate sepsis: 1/2 distal cecum was ligated with nylon suture (avoid ligating ileum and cecal mesenteric vessels). At the midpoint between the ligation and the end of the cecum, a 21 G sterile syringe needle was used to make a hole from the mesenteric edge to the mesenteric edge. Mouse model of severe sepsis: The distal cecum was ligated with nylon thread 3/4 to prevent accidental ligation of the cecal mesenteric vessels and ileum. A 20 G sterile syringe needle was used to puncture from the mesangial edge to the corresponding mesangial edge at the middle and ligated parts of the cecal end. After manually extruding part of the feces, the cecum was placed into the abdominal cavity and the abdominal tissues were sutured with 6-0 suture. A total of 1 mL sterile normal saline was injected subcutaneously at 37 °C [12]. Cecal perforation and ligation were not performed in the Sham group.

### 2.3. Experimental Procedures

The mice were randomly divided into 6 groups using a random number table: the sham group, the moderate CLP group, the moderate CLP + 2% H_2_ group, the moderate CLP + 67% H_2_ group, the severe CLP group, and the severe CLP + 67% H_2_ group (*n* = 60 per group). The CLP model was established by the cecal ligation and puncture (CLP) procedure in the CLP and CLP + H2 groups. H2 was administered by inhalation for 60 min in the CLP + H_2_ groups at 1 h and 6 h after the operation. The mice in the sham and CLP groups inhaled air only. The mice were sacrificed, and lung tissues were obtained from the different groups at 24 h after sham or CLP surgery. Moreover, for the different groups of mice (*n* = 20 per group), the 7-day survival was recorded.

### 2.4. Hydrogen Treatment

Inhalation of low concentration hydrogen: experimental mice were put into a sealed resin glass box (20 × 18 × 15 cm) with inlet and outlet, a GCH-500 high-purity hydrogen generator (Tianjin Spectral Analysis Instrument Technology Co., Tianjin, China) was used to generate hydrogen, and the air and hydrogen were premixed with a gas flowmeter; then, it was input into the sealed box at the speed of 4 L/min. The concentration of oxygen in the box was maintained at 33% through the gas detector, and the concentration of hydrogen in the box was detected by the hydrogen detector.

Inhalation of high concentration hydrogen: mice were in the same environment as above. AMS-H-01 hydrogen and oxygen atomizer (purchased from Shanghai Asclepius Meditec Co., Shanghai, China) was used to produce 67% hydrogen, which was input into the sealed box from the inlet through a special pipe. The hydrogen and oxygen mixture was input 30 min before the experiment to precharge the sealed box. A layer of calcium lime was laid on the bottom of the box to absorb the carbon dioxide gas produced by the breathing of the mice. Throughout the process, the hydrogen concentration in the box was continuously monitored by heat tracing gas analyzer (Thermal Fisher, Waltham, MA, USA). Mice in the hydrogen group inhaled hydrogen at the corresponding concentration for 1 h at 1 h and 6 h after CLP or the sham operation. Control group: mice were housed in the same environment, without hydrogen inhalation, with only air and oxygen inhalation, and the oxygen concentration was maintained at 33%.

### 2.5. Arterial Blood Gas Measurement

Mice were intubated arterially using a thinner cannula. After being immobilized, the mice were placed in a closed box and given either 2% or 67% hydrogen. After the hydrogen concentration was stable, the arterial blood of mice was drawn from the carotid artery by a trocar and analyzed by GEM Premier 3000 gas analyzer (Instrumentation Laboratory, Boston, MA, USA).

### 2.6. Detection of Pulmonary Myeloperoxidase (MPO) Activity

The blood of mouse lung tissue was washed with PBS solution, and the wet weight was weighed after the filter paper was sucked dry. A total of 10% homogenate was prepared by mixing normal saline, the MPO reaction was measured, and 590 nm of absorbance data were detected by the spectrophotometer (DU 640; Beckman, Miami, FL, USA).

### 2.7. Lung Wet-to-Dry Weight Ratio

The lung wet-to-dry weight ratio was used to represent the edema of lung tissue. The left lung of mice was taken and weighed with wet weight. The same lung tissue was placed in a microwave oven at 80 °C for 48 h, and the left lung was taken out and weighed with dry weight. The wet/dry weight ratio of the lung was calculated by measuring the wet weight and dry weight of the lung. The ratio of wet weight to dry weight was determined as the lung W/D value.

### 2.8. Bronchoalveolar Lavage and Determination of Protein Content

The mice were anesthetized and fixed. The chest of the mice was cut open. The trachea and lungs were exposed. The trachea was cut open and a catheter was inserted and fixed. A total of 0.3 mL PBS (pH 7.4) was injected into the lung through a catheter for 30 s, the lavage solution was extracted, and this injection and extraction process was repeated three times. The obtained alveolar lavage fluid was centrifuged at 1500× *g* r/min at 4 °C for 10 min to obtain the supernatant, which was stored in a refrigerator at −20 °C for measurement [11]. The protein content of alveolar lavage fluid was measured by BCA protein quantitative method.

### 2.9. Lung Histopathological Detection

Lung tissue was fixed in 4% paraformaldehyde for 24 h. The tissue was embedded in paraffin and cut into 8-µm-thick sections. After that, sections were conducted by hematoxylin and eosin (H&E) staining, and an ordinary optical microscope (BioRevo BZ-9000; Keyence, Osaka, Japan) was used to evaluate lung injury. With the guidance of pathologists, the scoring system of lung injury was carried out according to our previously published research [13].

### 2.10. Pathological Changes

Pathological changes were observed and evaluated with a double-blind reference to the relevant literature [14,15,16]. The pathological evaluation of the organ focused on hyperplasia, alveolar hemorrhage and consolidation, tissue infiltration, neutrophil migration, congestion and edema, and the degree was divided into none, mild, moderate and severe. Under the guidance and assistance of pathology experts, the organ pathology of the HE stained film was observed and evaluated according to relevant evaluation indexes.

### 2.11. Mitochondrial Morphology

The morphology of mitochondria was observed by transmission electron microscopy 24 h after CLP. Lung tissues were cut into 1-mm cubes, fixed with 2.5% glutaraldehyde, and stored at 48 °C for 24 h. The cubes were embedded in Spurr’s resin, cut into 0.12-mm-thick sections, and stained with 0.2% lead citrate and 1% uranyl acetate. The images were examined with a transmission electron microscope (JEM-1200X, Shimadzu, Kyoto, Japan).

### 2.12. Detection of Antioxidant Enzyme Activity

Lung tissue homogenate and serum were taken, and the reactions between the antioxidant enzyme catalase (CAT) and Superoxide dismutase (SOD) were measured by Du-640 ultraviolet spectrophotometer according to the test kit manufacturer’s instructions [15,16].

### 2.13. Enzyme-Linked Immunosorbent Assay (ELISA)

Mouse lung tissue homogenate and serum were taken, and the concentration of 8-iso-PGF2α and content of proinflammatory cytokine high mobility group box (HMGB1) (IBL, Munich, Germany) were detected by ELISA according to the test kit manufacturer’s instructions (Ann Arbor, MI, USA) [15,16].

### 2.14. Extraction of Mitochondria from Lung Tissue

Mice were sacrificed 24 h after CLP, and the lung tissues of mice were washed with the prepared low-temperature preservation PBS solution and dried. The chopped mushy lung tissue was transferred to a homogenizer with pipette (avoid left-right rotation to ensure the integrity of mitochondrial membrane). and fully homogenized with centrifugal tubes. The precooled centrifuge was centrifuged 1000× *g* at 4 °C for 10 min, and the lung tissue was filtered with double-layer gauze, leaving the supernatant. The filtrates were centrifuged again under the same parameters, and the precipitates were used as mitochondria.

### 2.15. Mitochondrial Respiratory Control Rate (RCR)

The isolated mitochondria (1 mg/mL) and 3 mL RCR medium were incubated at 30 °C constant temperature. The oxygen consumption rates (nmol of O_2_ min^−1^ mg protein^−1^) were evaluated with a Clark oxygen electrode (Hansatech Instruments Ltd., King’s Lynn, UK). The respiratory rate of state III and state IV were recorded according to the oxygen consumption curve. The ratio of state III (ST3) to state IV (ST4) was RCR.

### 2.16. Mitochondrial Membrane Potential (*Δ*Ψ m)

Mitochondrial membrane potential was measured by JC-1 kit (Beyotime, Shanghai, China) and detected by fluorescence spectrophotometer.

### 2.17. Mitochondrial Respiratory Chain Complex Activity

Respiratory chain complex I activity assay: 50 μg (0.5 mL) mitochondrial protein was added into the buffer system with antimycin concentration of 2 μg/mL and NADH concentration of 0.13 mm. Light absorption was detected at 340 nm and left for about 1 min. A total of 0.065 mM CoQ was added and mixed evenly, reacted, absorbance change was detected, and left for about 4 min. A total of 2 μg/mL rotenone was added and reacted for about 3 min. The strongest rotenone response may reflect the activity of NADH ubiquinone oxidoreductase. The reaction of chain absorbion complex enzyme I can be represented by the decline rate of NADH within 1 min, with an extinction coefficient of E = 6.81/mM/cm.

Determination of respiratory chain complex II activity: 5–10 μg mitochondrial protein and 20 mM sodium succinate were added to the buffer solution and maintained at 30 °C for 10 min. A total of 0.05 mm DCPIP, 2 μg/mL rotenone and 2 μg/mL antimycin A were addd, mixed evenly for 3 min, and baseline absorbance changes were detected at 600 nm. A total of 0.065 mM ubiquinone was added to initiate the reaction, and the absorbance change was detected for about 4 min. The content change of DCPIP was detected as a respiratory chain complex enzyme II reaction. The DCPIP extinction coefficient E = 21/mM/cm.

### 2.18. Western Blot

Equal amounts of protein from cells were separated by SDS–PAGE (10% polyacrylamide gels) and electrotransferred to nitrocellulose. Membranes were blocked with 5% defatted milk in Tris-buffered saline, pH 7.6, containing 0.1% (*v*/*v*) Tween 20 (TBST). Membranes were incubated with primary antibodies at 4 °C, total Drp1 1:1000 (ab184247, Abcam, Cambridge, MA, USA), Mfn2 1:1000 (ab56889, Abcam, Cambridge, MA, USA), β-actin 1:1000 (Sigma, St. Louis, MO, USA) and re-blotted with corresponding horseradish peroxidase-linked secondary antibody 1:5000 (Proteintech Group, CHI, IL, USA). The bands were detected using ECL (Perkin Elmer, Waltham, MA, USA) with an exposure to Kodak film and quantified by scanning densitometry. The protein content was normalized by β-actin.

### 2.19. Statistical Analysis

Through SPSS 24.0 (Version X; IBM, Armonk, NY, USA), the measurement data were expressed by Mean ± SD, and the survival rate was expressed by percentage. The measurement data were analyzed by one-way ANOVA, and different groups were compared by LSD-*t* test. Survival rates were compared by Fisher’s deterministic probability method. *p* < 0.05 represents a statistically significant difference.

## 3. Results

### 3.1. Hydrogen Inhalation Did Not Cause Hypoxia in Mice

By measuring the arterial blood gas after the input hydrogen concentration was stable for 30 min, the arterial blood PaCO_2_, PaO_2_ and pH values of mice in each group were monitored. There was no significant difference between the arterial blood of each group (Table 1), which indicated that mice did not suffer from hypoxia during the inhalation of 2% and 67% hydrogen.

### 3.2. Both 2% and 67% Hydrogen Inhalation Could Improve Sepsis in Mice

**Figure 1:**
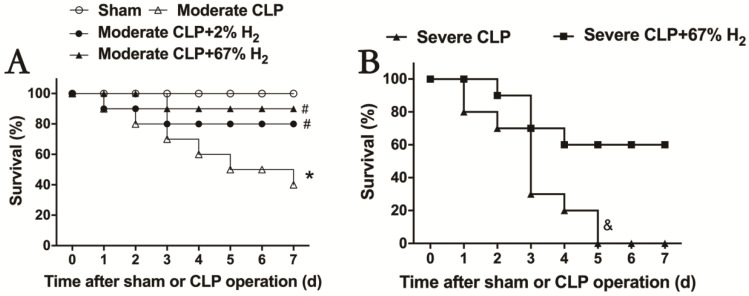
Hydrogen inhalation improved the survival rate of sepsis mice. (**A**) 2% and 67% hydrogen inhalation could significantly improve the 7-day survival rate of sepsis mice. (**B**) 67% hydrogen inhalation improved the survival rate of severe sepsis mice. * *p* < 0.05 vs. Sham group. # *p* < 0.05 vs. moderate CLP group. & *p* < 0.05 vs. Severe CLP group.

After hydrogen treatment, the 7-day survival rate of septic mice was recorded, and the therapeutic effects of different concentrations of hydrogen inhalation on moderate and severe sepsis were observed (Figure 1). The 7-day survival rate of mice with moderate sepsis is 40% (*p <* 0.05, Moderate CLP vs. Sham), while that of moderate sepsis mice treated with 2% hydrogen inhalation increased to 80%; meanwhile, the survival rate of moderate sepsis mice treated with 67% hydrogen inhalation can reach 90%. There was significant difference in the improvement of survival rate in hydrogen group compared to CLP group (*p <* 0.05, Moderate CLP + 2% H_2_ vs. Moderate CLP; *p <* 0.05, Moderate CLP + 67% H_2_ vs. Moderate CLP) (Figure 1A). Inhalation of 67% hydrogen could increase the 7-day survival rate of severe sepsis animals from 0% to 60% (*p <* 0.05, Severe CLP + 67% H_2_ vs. Severe CLP) (Figure 1B). The above data demonstrated that hydrogen inhalation could significantly improve the 7-day survival rate of septic mice, and had a certain concentration effect.

### 3.3. A Total of 67% Hydrogen Could Significantly Reduce Sepsis-Induced Organ Injury Compared with 2% Hydrogen

**Figure 2:**
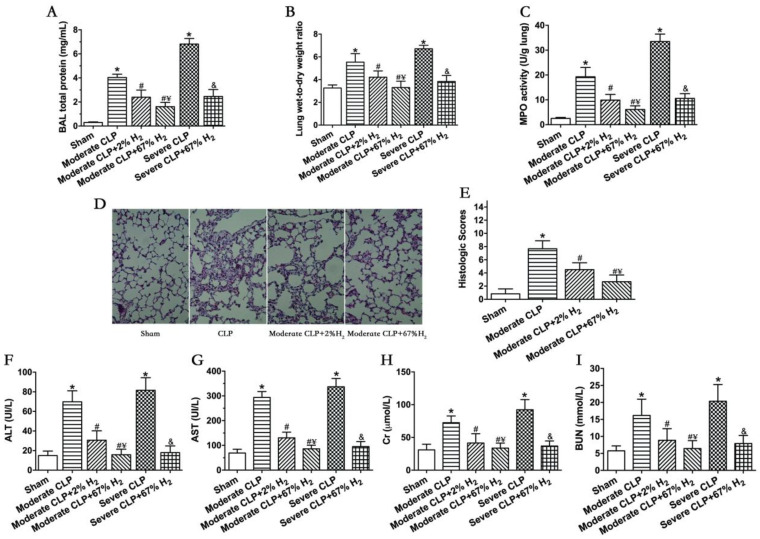
A total of 67% and 2% H_2_ inhalation attenuated organ injury in septic mice model. Data were monitored and recorded at 24 h after CLP or sham operation. (**A**) Lung BAL total protein; (**B**) Lung wet-to-dry weight ratio; (**C**) Lung MPO activity; (**D**) HE staining of lungs (400×); (**E**) Lung histologic scores; Liver injury was represented by (**F**) ALT and (**G**) AST; Kidney injury was indicated by (**H**) Cr and (**I**) BUN. Values are expressed as means ± SEM (*n* = 6 per group). * *p* < 0.05 vs. Sham group. # *p* < 0.05 vs. Moderate CLP group. ¥ *p* < 0.05 vs. Moderate CLP + 2% H_2_ group. & *p* < 0.05 vs. Severe CLP group.

Septic mice showed significant pulmonary dysfunction 24 h after the operation (Figure 2). Compared with Sham group, the protein content in broncholavage fluid, lung wet-to-dry ratio and lung MPO activity of mice in CLP group increased significantly (*p* < 0.05, Moderate CLP vs. Sham; *p <* 0.05, Severe CLP vs. Sham). Compared with the Moderate CLP group, 2% and 67% hydrogen inhalation could reduce the increase in the above indexes (*p <* 0.05, Moderate CLP + 2% H_2_ vs. Moderate CLP; *p <* 0.05, Moderate CLP + 67% H_2_ vs. Moderate CLP), and 67% hydrogen inhalation exert a stronger effect on improving moderate sepsis-induced acute lung injury than 2% hydrogen (*p* < 0.05, Moderate CLP + 67% H_2_ vs. Moderate CLP + 2% H_2_). At the same time, 67% hydrogen inhalation can alleviate the increase in lung injury indexes in severe septic mice compared with the Severe CLP group (*p <* 0.05, Severe CLP + 67% H_2_ vs. Severe CLP) (Figure 2A–C). There are pathological changes in lung of sepsis mice, such as bleeding, consolidation, alveolar and pulmonary interstitial neutrophil infiltration, alveolar wall thickening and so on. Compared to CLP group, 2% and 67% hydrogen inhalation can improve the lung pathological changes and reduce the histological score of moderate sepsis mice (*p <* 0.05, Moderate CLP + 67% H_2_ vs. Moderate CLP group; *p <* 0.05, Moderate CLP + 2% H_2_ vs. Moderate CLP) (Figure 2D,E). Meanwhile, 2% and 67% hydrogen inhalation could significantly improve the indexes of liver and kidney function injury in moderate CLP (*p* < 0.05, Moderate CLP + 2% H_2_ vs. Moderate CLP; *p* < 0.05, Moderate CLP + 67% H_2_ vs. Moderate CLP), and 67% hydrogen inhalation had better protective effect (*p* < 0.05, Moderate CLP + 67% H_2_ vs. Moderate CLP + 2% H_2_); Compared with the severe sepsis group, high concentration hydrogen inhalation significantly improved liver and kidney function injury in severe septic mice (*p <* 0.05, Severe CLP + 67% H_2_ vs. Severe CLP) (Figure 2F–H).

### 3.4. High Concentration of Hydrogen Could Reverse the Imbalance between Inflammation and Oxidative Redox

**Figure 3:**
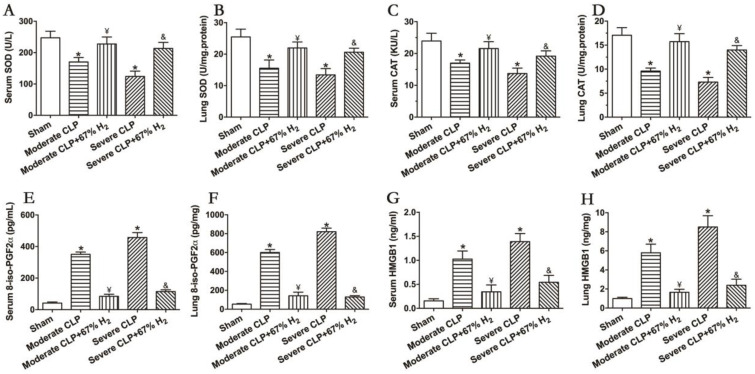
67% H_2_ inhalation improved the activities of SOD and CAT, and down-regulated levels of 8-iso-PGF2α HMGB1 and HMGB1. (**A**,**B**) Serum and lung SOD activity; (**C**,**D**) Serum and lung CAT activity; (**E**,**F**) Serum and lung 8-iso-PGF2α level; (**G**,**H**) Serum and lung HMGB1 level. The blood and lungs were sampled for testing these indicators at 24 h after CLP or sham operation. All the data were expressed by mean ± SEM (*n* = 6 per group). * *p* < 0.05 vs. Sham group. ¥ *p* < 0.05 vs. Moderate CLP group. & *p* < 0.05 vs. Severe CLP group.

The activities of antioxidant enzymes SOD and CAT can reflect the antioxidant stress response in vivo. The results demonstrated that the activities of the two antioxidant enzymes decreased significantly after the CLP surgery (*p <* 0.05, Moderate CLP vs. Sham; *p <* 0.05, Severe CLP vs. Sham), and 67% hydrogen absorption can significantly improve the activities of CAT and SOD in moderate and severe septic mice (*p* < 0.05, Moderate CLP + 67% H_2_ vs. Moderate CLP; *p <* 0.05, Severe CLP + 67% H_2_ vs. Severe CLP) (Figure 3A,B). Meanwhile, 8-iso-PGF2α was detected to demonstrate the oxidative metabolic response. The content of oxidation product 8-iso-PGF2α in the serum and lungs of animals with moderate and severe sepsis increased significantly, and there was a significant difference compared with the Sham group (*p <* 0.05, Moderate CLP vs. Sham; *p <* 0.05, Severe CLP vs. Sham), while 67% of the hydrogen inhalation treatment could significantly reduce the content of the oxidation product 8-iso-PGF2α (*p* < 0.05, Moderate CLP + 67% H_2_ vs. Moderate CLP; *p <* 0.05, Severe CLP + 67% H_2_ vs. Severe CLP) (Figure 3C). It is well known that the proinflammatory cytokine HMGB1 plays an important role in the pathogenesis of sepsis. We detected it and found that the expression of HMGB1 in lungs and serum of mice with moderate and severe sepsis increased significantly (*p <* 0.05, Moderate CLP vs. Sham; *p <* 0.05, Severe CLP vs. Sham), while 67% of the hydrogen inhalation treatment can significantly reduce the content of HMGB1 of septic mice (*p* < 0.05, Moderate CLP + 67% H_2_ vs. Moderate CLP; *p <* 0.05, Severe CLP + 67% H_2_ vs. Severe CLP) (Figure 3D). The above results demonstrate that 67% hydrogen can reverse the imbalance between inflammation and oxidative stress.

### 3.5. A Total of 67% Hydrogen Reduced the Degree of Mitochondrial Damage

**Figure 4:**
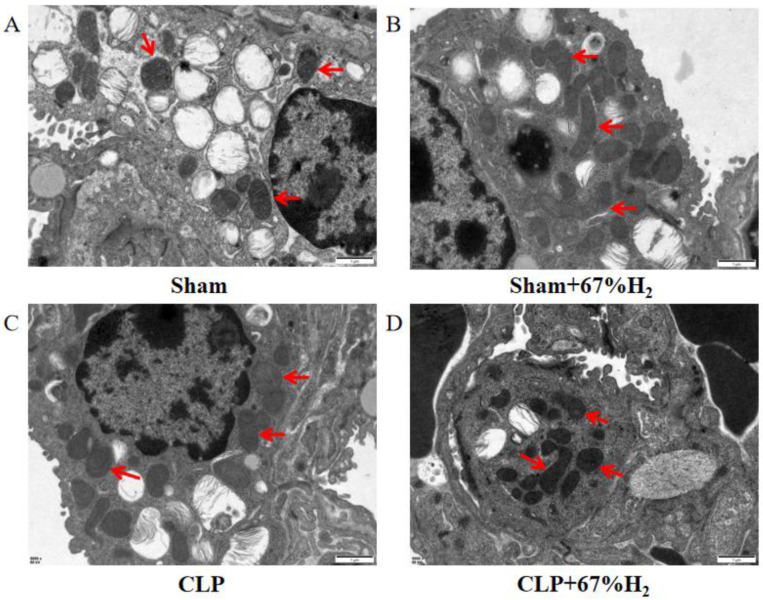
A total of 67% H_2_ inhalation reduced the degree of mitochondrial damage. As shown by the red arrows, (**A**,**B**) Mitochondrial ridges were intact without swelling. (**C**) Mitochondria became vacuolated and no mitochondrial ridges were observed. (**D**) Part of the mitochondrial ridges disappeared and the mitochondria were slightly swollen.

We observed the morphology of lung mitochondria via TEM. In the Sham and Sham + H_2_ groups, the mitochondrial ridges were intact and showed no obvious swelling (Figure 4A,B), whereas the mitochondria in the CLP group underwent vacuolation and exhibited no mitochondrial ridges (Figure 4C). These changes were mitigated in the CLP + 67% H_2_ group (Figure 4D).

### 3.6. A Total of 67% Hydrogen Treatment Could Maintain Mitochondrial Structural Integrity

**Figure 5:**
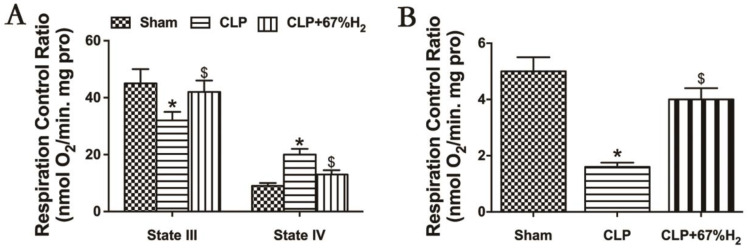
Hydrogen treatment could maintain mitochondrial structural integrity. State III: oxygen consumption rate under ADP presence. State IV respiration rate: the oxygen consumption rate with ADP exhausted. RCR is the value of state III/state IV. (**A**) State III and IV respiration rate; (**B**) RCR. Values are expressed as mean ± SD (*n* = 6). * *p <* 0.05 versus the Sham group. $ *p <* 0.05 versus CLP group.

We hypothesized that hydrogen gas may play a protective role in sepsis by ameliorating mitochondrial dysfunction. RCR reflects the structural integrity of mitochondria and the coupling degree of oxidative phosphorylation performed by mitochondria [17], and its calculation method is the respiratory rate of state III respiration/the respiratory rate of state IV respiration. Sepsis-induced acute lung injury decreased the mitochondrial state III respiration (ST3) and increased the state IV respiration (ST4) in CLP group (*p* < 0.05, CLP vs. Sham), but high concentration hydrogen inhalation decreased mitochondrial ST4 and increased ST3 (*p* < 0.05, CLP + 67% H_2_ vs. CLP) (Figure 5A). Compared with the sham operation group, the mitochondrial RCR of CLP group decreased significantly (*p* < 0.05, CLP vs. Sham). The RCR of CLP + H_2_ group was notably higher than that of CLP group (*p* < 0.05, CLP + 67% H_2_ vs. CLP), indicating that the mitochondrial respiratory function of the lung tissue of septic mice was improved after high concentration hydrogen inhalation (Figure 5B).

### 3.7. A Total of 67% Hydrogen Significantly Ameliorated Sepsis-Induced Mitochondrial Dysfunction

**Figure 6:**
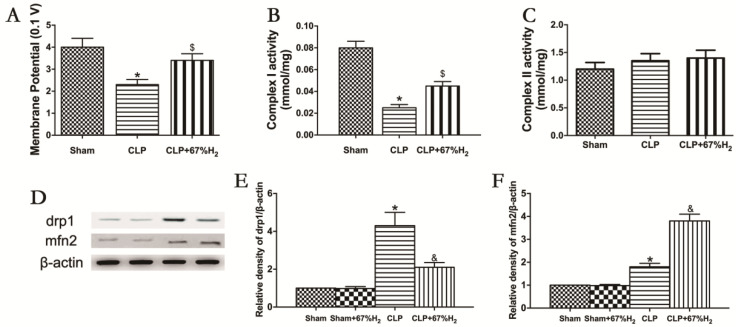
H_2_ ameliorated sepsis-induced mitochondrial dysfunction. (**A**) MMP; (**B**) Complex I activity; (**C**) Complex II activity; (**D**) The expression of Drp1 and Mfn2 was detected by Western blot analysis; quantitative analysis of (**E**) Drp1 and (**F**) Mfn2 are presented as the ratio of band density to that of β-actin. Data were output as mean ± SD (*n* = 6). * *p <* 0.05 vs. Sham group. $ *p <* 0.05 vs. CLP group. & *p* < 0.05 vs. CLP group.

Mitochondrial function was assessed by measuring MMP, mitochondrial respiratory chain complex activities, and expression of fusion and fission proteins. Compared with the Sham group, MMP in CLP group and CLP + H_2_ group decreased significantly (*p* < 0.05, CLP vs. Sham; *p <* 0.05, CLP + H_2_ vs. Sham), suggesting that sepsis may reduce mitochondrial membrane potential, which cannot be completely improved even by hydrogen inhalation. MMP in the CLP + H_2_ group was significantly higher than that in the CLP group (*p* < 0.05, CLP + 67% H_2_ vs. CLP) (Figure 6A). The activity of the mitochondrial respiratory chain complex I in lung tissue decreased significantly in CLP group (*p* < 0.05, CLP vs. Sham), but there was no significant change in the activity of the mitochondrial respiratory chain complex II in the CLP mouse model compared with the control group (*p* > 0.05, CLP vs. Sham), indicating that sepsis may damage the activity of mitochondrial respiratory chain complex I in lung tissue, but had no significant effect on the activity of complex II. Hydrogen inhalation could significantly improve the activity of the mitochondrial respiratory chain complex I (*p* < 0.05, CLP vs. Sham) (Figure 6B,C). The expression of Drp1 protein in CLP group was notably higher than that in the Sham group, while the expression of Mfn2 significantly decreased (*p* < 0.05, CLP vs. Sham). Compared with the CLP group, the expression of Drp1 protein decreased and the expression of Mfn2 protein dramatically increased in the CLP + H_2_ group (*p* < 0.05, CLP vs. Sham) (Figure 6D–F). The results demonstrated that high concentration hydrogen inhalation could improve the MMP, improve the fusion of the mitochondria and reduce mitochondrial fission, thus improving the mitochondrial function of the lung tissue of septic mice.

## 4. Discussion

Because sepsis is a very complex pathophysiological process, studying the mechanism and treatment in critically ill septic patients is challenging and has limitations. As a result, investigations on animal sepsis models can provide additional insight into this critical topic. The CLP model used in this experiment is considered as the gold standard in sepsis research, which can ideally simulate the in vivo changes of sepsis in clinical patients [18]. The 7-day survival rate is generally 30% to 40% in moderate sepsis mice, and 0% in severe sepsis mice. In addition, with the progress of the disease course, moderate and severe sepsis mice demonstrated different increases in mortality and different degrees of organ damage (lung, liver and kidney) accompanied by obvious organ dysfunction, which demonstrated that the CLP sepsis mouse model was established successfully in this study (Figure 1 and Figure 2). This experiment demonstrated that hydrogen inhalation treatment can increase the survival rate of moderate and severe sepsis, reduce the level of organ dysfunction in animals, and significantly regulate tissue function, which indicates that hydrogen inhalation can be an effective treatment for sepsis.

Hydrogen exists widely in nature, and is colorless and odorless. It is a diatomic gas molecule with the lowest molecular weight among all gases known to mankind. High concentration hydrogen significantly improves the survival rate of septic mice in this study; due to its inhalation through the respiratory tract, the protective effect of high pressure hydrogen on lung tissue injury should be better than that of low concentration hydrogen. Hydrogen has significant therapeutic effects on the dysfunction of organs such as intestine, kidney, liver, lung, myocardium and brain. Not only that, it also has definite therapeutic effect on type 2 diabetes, multiple organ injury, sepsis, atherosclerosis, tumor and many other diseases [19,20,21,22,23,24]. At present, we know that hydrogen is insoluble in water. After entering the circulation through the alveolar epithelium, there are few components dissolved in the blood, and no binding effect with other components in the blood is found. Therefore, most of it is discharged from the body in the same form as it entered the body. In this study, the arterial blood gas of septic mice was analyzed after hydrogen inhalation. There was no significant difference in arterial blood partial pressure and blood oxygen saturation between high concentration hydrogen inhalation group and low concentration hydrogen inhalation group. In addition, the mice in this analysis did not show hypoxia during the test.

During sepsis, the changes of biochemical factors lead to the imbalance of the redox system and inflammatory response, leading to the formation of peroxidation in the body, which will strengthen the response of the systemic inflammatory response syndrome (SIRS) [25]. The excessive reactive oxygen species not neutralized in the body can cause oxidative damage to body tissues, and reactive oxygen species can cause damage to DNA, protein and lipid [26]. This imbalance of peroxidation is related to the decrease in plasma and tissue levels of antioxidants [such as glutathione (GSH), thioredoxin and selenium (antioxidant enzymes and required for GSH production)], as well as the decrease in mitochondrial ATP level and the presence of oxidized lipids. In clinical trials of sepsis, the addition of glutamine (a precursor of GSH) and/or selenium to parenteral nutrition can improve clinical outcomes and reduce the intensity of SIRS response [27]. This suggests that there may be a causal relationship between the redox imbalance, SIRS response intensity and other downstream events during sepsis. During the inflammatory response, activated neutrophils and macrophages produce a large amount of reactive oxygen species (ROS) and, in some cases, reactive nitrogen (RNS) [28]. Neutrophil MPO produces H_2_O_2_ derivatives (such as hypochlorous acid), and hydroxyl radical (HO•) is the most reactive of all oxygen radicals. ROS and RNS products can lead to reversible or irreversible chemical changes (oxidation, nitration and nitrosation) in proteins, lipids and DNA, resulting in decreased biochemical function [29]. ROS and RNS can induce DNA adducts, leading to DNA breakage. In general, excessive ROS and RNS products produced during sepsis can damage cells and organs [30]. The concentrations of ROS and RNS in a steady state are strictly regulated by a series of inducible antioxidant enzymes. Important antioxidant enzymes include CAT and SOD. The activities of them in serum and organ tissues can reflect the level of antioxidants and represent the antioxidant damage ability of the body. 8-iso-PGF2α is a reliable indicator reflecting the degree of oxidative stress [31,32]. The level of HMGB1 is closely related to the prognosis of sepsis. It is an important member of the alarm hormone in vivo [33]. As a typical alarm protein [34], the HMGB1 can induce inflammation after transfer to the outside. In this study, the activity of CAT and SOD in organs and serum of moderate and severe sepsis animals decreased significantly, and the content of oxidation reaction product 8-iso-PGF2α increased significantly. These changes can be improved by hydrogen inhalation treatment. We also found that HMGB1 increased significantly in sepsis model mice and decreased significantly after hydrogen treatment. The increase in HMGB1 in septic mice is the increase in the body’s response to external harmful factors, which is consistent with the previous research results [35], which means that the therapeutic effect of hydrogen on sepsis may be related to the oxidation reaction system in the animal body.

Mitochondria are organelles with bilateral membrane structure. Their composition includes more than 1000 protein, lipid molecules and a genome. They can perform biological functions under the dual regulation of their own genes and nuclear genes, which makes them the most important organelle in eukaryotic cells. Mitochondria are in the process of continuous movement and fusion/fission according to the changes of cell function. They are the respiratory organ and energy metabolism center of the cells. They can perform the energy metabolism process of a variety of substances and produce numerous ATP to provide basic and necessary energy for animal activities. They are also the convergence center of multiple signal pathways related to innate immunity, because they are the main site of ROS production in cells. Their dysfunction can lead to cell apoptosis, which causes a variety of diseases, including Alzheimer’s and other neurodegenerative diseases, mental diseases (depression), muscle diseases, cancer and so on [36,37,38,39,40,41]. In this study, the acute lung injury model was prepared by CLP to simulate the pathogenesis of sepsis. The production of ROS affects energy generation and the stability of all cell activities, which is due to the opening of the inner membrane anion channels, the opening of the mitochondrial permeability transition pore and membrane channels, lipid peroxidation, changes in mitochondrial DNA, etc. Moreover, the accumulation of ROS in the mitochondria impairs organelle function [42,43]. The opening of these channels leads to the collapse of its membrane potential and the instantaneous increase in ROS produced by electron transport chain, which is called the ROS-induced ROS release (RIRR) [44]. MMP is a prerequisite for oxidative phosphorylation and ATP synthesis, and its decline is a sign of impaired mitochondrial function. In this study, the hydrogen inhalation treatment can significantly change the level of mitochondrial membrane potential in septic mice, which plays an important role in maintaining its normal function. The three proton pumping complexes of the electron transfer chain are NADH-ubiquinone oxidoreductase or complex I, ubiquinone cytochrome C oxidoreductase or complex III, and cytochrome C oxidase or complex IV. Succinate dehydrogenase or complex II does not pump protons, but provides reduced ubiquinone. This study demonstrated that hydrogen exerted a role by improving the activity of mitochondrial respiratory chain complex I of septic mice, and its possible mechanism was to reduce the damage of reactive oxygen species free radicals to the respiratory chain complex.

The structure of mitochondria is highly dynamic, and its shape is specific according to different cell types. Mitochondrial morphological change is achieved by regulating its dynamic characteristics, including fusion, fission, movement and position tethering. Mitochondrial dynamics also plays an important role in a variety of cellular signaling pathways, and many mechanisms also play a role in regulating its structure [45]. Mitochondria regulate their structure and function through fusion and fission and autophagy. When fusion and fission are relatively balanced, ATP production meets cell needs, membrane potential is high, and mitochondria remove damaged organelles through autophagy. Fusion can be achieved by increasing the expression of fused dynamin-related proteins (DRPs), increasing the oligomerization of Mfn2, stabilizing OPA1 long, short or down regulating Drp1-dependent mitotic activity [46]. Highly conserved proteins that mediate its fission and fusion are members of the DRP family [47,48], and these proteins are larger self-assembled GTPases. Mitochondrial mitotic protein, Drp1, mediates the membrane rupture. DRP constitutes the core of the fusion and fission machinery and is the target of various regulatory mechanisms, including the regulation of steady-state expression, proteolytic processing and post-translational modification [49]. Genetic and chemical inhibition of Drp1 will increase the MMP and oxidative phosphorylation of larger cells in the population, making mitochondria more active in large cells, but not in small cells [50]. In this experiment, the expression of Drp1 protein in the CLP group was higher than that in Sham group, while the expression of Mfn2 decreased. Compared with CLP group, Drp1 protein expression was decreased and Mfn2 protein expression was increased in CLP + H_2_ group, indicating that lung injury of septic mice affected mitochondrial fusion and fission. Hydrogen inhalation treatment could improve fusion, reduce fission and enhance mitochondrial function.

## 5. Conclusions

Briefly, both high concentration hydrogen inhalation and low concentration hydrogen inhalation could improve the survival rate and organ injury of moderate and severe sepsis, and significantly reduced the injury of liver, kidney and lung tissues. The therapeutic effect of the high concentration hydrogen was better than that of the low concentration hydrogen. High concentration hydrogen inhalation could significantly alleviate the mitochondrial dysfunction of lung tissue induced by sepsis, and acted by affecting mitochondrial dynamic function.

## Figures and Tables

**Table 1:** Arterial blood gas of mice in each group after inhalation of 2% and 67% hydrogen.

Group	pH	PaO_2_	PaCO_2_
Sham	7.41 ± 0.11	96.52 ± 3.16	35.81 ± 1.37
Sham + 2% H_2_	7.40 ± 0.13	96.45 ± 2.96	35.77 ± 1.58
Sham + 67% H_2_	7.40 ± 0.14	95.83 ± 3.79	36.04 ± 1.60
Moderate CLP	7.39 ± 0.16	95.89 ± 3.76	35.38 ± 1.53
Moderate CLP + 2% H_2_	7.41 ± 0.17	96.83 ± 3.61	36.29 ± 1.70
Moderate CLP + 67% H_2_	7.39 ± 0.16	95.18 ± 3.68	36.90 ± 1.64
Severe CLP	7.39 ± 0.22	95.78 ± 3.80	35.40 ± 1.86
Severe CLP + 2% H_2_	7.38 ± 0.25	95.86 ± 3.96	37.28 ± 1.69
Severe CLP + 67% H_2_	7.40 ± 0.28	95.67 ± 4.01	37.12 ± 1.92
